# A Novel Hierarchical Security Solution for Controller-Area-Network-Based 3D Printing in a Post-Quantum World

**DOI:** 10.3390/s23249886

**Published:** 2023-12-17

**Authors:** Tyler Cultice, Joseph Clark, Wu Yang, Himanshu Thapliyal

**Affiliations:** Department of Electrical Engineering and Computer Science, The University of Tennessee, Knoxville, TN 37996, USA; tcultice@vols.utk.edu (T.C.); jclar168@vols.utk.edu (J.C.); wyang19@vols.utk.edu (W.Y.)

**Keywords:** cybersecurity, Controller Area Network, post-quantum cryptography, lightweight cryptography, additive manufacturing, 3D printing

## Abstract

As the popularity of 3D printing or additive manufacturing (AM) continues to increase for use in commercial and defense supply chains, the requirement for reliable, robust protection from adversaries has become more important than ever. Three-dimensional printing security focuses on protecting both the individual Industrial Internet of Things (I-IoT) AM devices and the networks that connect hundreds of these machines together. Additionally, rapid improvements in quantum computing demonstrate a vital need for robust security in a post-quantum future for critical AM manufacturing, especially for applications in, for example, the medical and defense industries. In this paper, we discuss the attack surface of adversarial data manipulation on the physical inter-device communication bus, Controller Area Network (CAN). We propose a novel, hierarchical tree solution for a secure, post-quantum-supported security framework for CAN-based AM devices. Through using subnet hopping between isolated CAN buses, our framework maintains the ability to use legacy or third-party devices in a plug-and-play fashion while securing and minimizing the attack surface of hardware Trojans or other adversaries. The results of the physical implementation of our framework demonstrate 25% and 90% improvement in message costs for authentication compared to existing lightweight and post-quantum CAN security solutions, respectively. Additionally, we performed timing benchmarks on the normal communication (hopping) and authentication schemes of our framework.

## 1. Introduction

Three-dimensional printing is a rapidly growing industry, receiving massive support from consumers and industries alike. The demand for additive manufacturing (AM) services and goods has brought forth huge revolutions in 3D printing technologies as industries develop networks of 3D printing systems, all centered around the ideas of the RepRap movement. Modern 3D printing systems highlight detailed networking capabilities, complex operating systems and automation, and a significant number of interconnected embedded systems and cyber–physical devices. The internal networks connecting various micro-controllers, sensors, etc., transmit critical information, such as coordinates and safety statuses, through physical communication protocols and interconnections. One such protocol that has seen growth in the market for 3D printers is the Controller Area Network (CAN) [1], which provides an error-correcting, high-interconnectivity method for fast transmission between several internal and external components of the printers.

However, the CAN protocol (and many others) provides only a foundation for communication without considerations for the security or integrity of the data transmitted; while much work has gone into the networking of PC-to-printer (external) communications, little security is typically provided to secure the internal structure of the printer from compromised modules, hardware Trojans, or other adversarial connections on the CAN bus. Because these buses connect the peripherals to the heart of the printer, the transmission of malicious data risks the operability, safety, and privacy of the systems and the users operating them. Modularity and compatibility have contributed significantly to the success of 3D printing as the AM community and industries utilize modified or custom-constructed parts to suit the needs of their applications. This opens up a wide attack surface for adversaries to exploit compromised/hacked 3D printing modules and connections which would provide unrestricted access to unsecured communication. This is a well-known issue for CAN bus applications in the vehicular industry [2], and recently has also been exploited and demonstrated on commercial additive manufacturing systems [3]. Attackers on CAN buses can eavesdrop, manipulate, block, and fabricate messages pertaining to safety-critical, telemetric, and intellectual property (or other private) information to and from all devices utilizing the CAN bus. For example, an attacking compromised node may at any time transmit a command to overheat the heater cartridge to critically unsafe temperatures. It has been shown in other applications how malicious alterations to commands and data like this attack can result in severe damage and safety risks to both users and property through other forms of attack [4]. Furthermore, denial-of-service (DoS) of the CAN bus would result in disabling most, if not all, devices’ connectivity to the central CPU as the bus controls all communication between the devices.

This issue is likely to become more significant in the coming years as 3D printing farms and systems rely on increasingly many interconnected, variably sourced parts with unknown supply chain security to operate in parallel and keep up with product demand. Many software packages, including the popular Ultimaker Cura, are capable of remotely operating and monitoring sensors, cameras, and other functions of multiple printers simultaneously, which requires more internal components to transmit even more data over the insecure communication protocols. Each new function and degree of connectivity increases the potential attack surface of Industrial Internet of Things (I-IoT)-enabled systems. CAN has also been used prior in communication systems for printer-to-PC connections, operating cameras, safety equipment, and more [5], widening the scope of these vulnerabilities and concerns. Additionally, the development of Shor’s Algorithm [6] and Grover’s Algorithm [7] have begun a theoretical countdown timer on the modern asymmetric and symmetric encryption and key generation schemes used in many typical or lightweight applications. This post-quantum future should also be factored into the security of critical infrastructure, especially when static, long-term keys are utilized for encryption and authentication purposes (such as server keys).

Three-dimensional printing security is difficult to design for as attacks can be launched using many attack surfaces both internal and external to the printer. A singular security solution, such as adding encryption to external connections of the 3D printer central CPU, is not sufficient to successfully cover the cyber–physical devices and independent systems within the printer that can become compromised or hold Trojan payloads. Successful attacks on these components can also easily jeopardize all other genuine devices operating within that system. Furthermore, as a core value of 3D printing is compatibility and modularity, it cannot be assumed that the supply chain for 3D printer components is always trustworthy. Thus, these systems must focus their efforts on securing the critical communication that connects, interprets, and uses messages potentially transmitted by adversary-compromised parts. However, manufacturers do not need to turn away from using CAN or networking capabilities within 3D printing and thus from the associated benefits. Instead, strong cyber–physical and data security systems must be designed and implemented while avoiding any major deviations from the CAN protocol to allow for maximum compatibility. To overcome these challenges, we propose a novel solution for 3D printing security with CAN.

### Contribution

**Figure 1:**
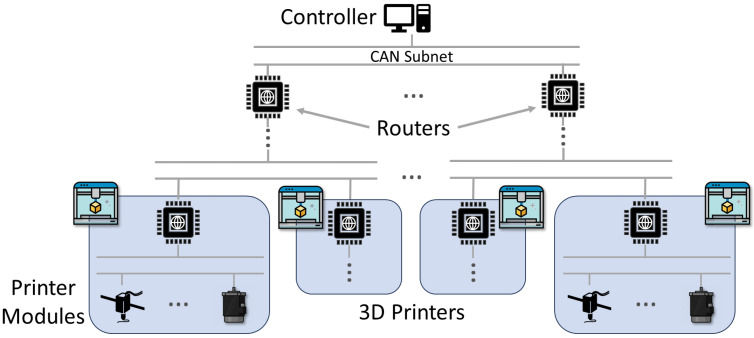
Structure of our CAN-based 3D printing framework based on a hierarchical root-of-trust structure. This provides a high-security network for 3D printers to utilize internally and externally to connect a large number of untrusted components.

In this work, we propose a novel, low-overhead security framework that restructures the CAN network to divide and isolate critical components, authenticate and validate messages, authenticate connected devices, and encrypt and obfuscate data from attackers. Unlike existing solutions, our framework is based on a hierarchical tree structure with root-of-trust originating from the root node (or PC), improving our previous CAN 3D printer work [8]. To realize this design, strictly authenticated devices called “routers” forward authenticated, encrypted messages from untrusted 3D printer devices, or “endpoints”. Existing security solutions revolve around the need for full trust to be established in devices before allowing them to connect at all to the system. Our framework allows a large array of untrusted 3D printer components to be connected while mitigating the potential damage and impact of adversarial devices, including denial of service attacks. This design is versatile and can support a range of cryptographic suites, from lightweight (LWC) to post-quantum cryptography (PQC), which is explored later in this work. Figure 1 demonstrates the overall structure of the framework utilized within an example 3D printer setup.

The novel contributions of our design can be summarized as follows:

- A novel root-of-trust-based 3D printing security scheme at the cyber–physical communications level.
- Proven efficient and effective post-quantum security for 3D printers with better performance than existing solutions and compatibility support for lightweight cryptographic algorithms.
- Support for a highly expandable array of 3D printers and 3D printer components with strong mitigation of attacks correlated with the supply chain and hardware Trojans (untrustworthy 3D printing components).
- Mitigation of denial-of-service (DoS) attacks via bus filtering methods on individual, isolated buses organized in a flexible tree design.

The organization of this work is as follows: Section 2 explains our vision and goals for hierarchical networks and cryptography as an AM security solution; Section 3 details background information and prior related work on CAN and 3D printing security; Section 4 details the proposed framework and its sub-components and operations; Section 5 analyzes the security, compatibility, and other considerations of the framework; Section 6 provides results from an implementation of the framework and compares them to existing CAN security solutions; finally, Section 7 concludes the paper and provides final thoughts.

## 2. Visions and Goals for a Secure 3D Printing Framework

Typical approaches to CAN security center around acceptance of only strongly authenticated endpoints and additional infrastructure, such as a “server”; while these conventional approaches work within their respective domains, 3D printers also include frequent component swapping and 3rd party supply chains, which severely restrict the ability for keys and authentication methods to be consistently reprogrammed within each device. For example, reprogramming the root node’s secure memory with new authentication keys for each endpoint each time new devices are connected may seriously weaken security against a malicious man-in-the-middle actor. Additionally, typical 3D printer security designs target the connections outside of the printer, such as data transfers or insecure storage of prepared 3D models; while these approaches are very effective at securing the external connections to the printer, the firmware and devices within the printer remain a huge target for adversaries.

Alongside strong security, 3D printer communications must also remain fast enough to continuously stream commands, safety information, and sensor data without severe bottlenecks; while algorithmic speed is not of critical importance, security solutions must take care to keep message transmission reactive and fast. Thus, there are three goals to focus on when designing 3D printer security for the internalized communication modules:

1. Maintain high security while upholding modularity and compatibility;
2. Minimize communication overhead;
3. Minimize changes to existing protocols.

First, a strong level of security that encompasses and protects as much of the system as possible is key to any security solution. Minimizing the damage that an adversary can do is an important goal for 3D printing security frameworks, including protection from denial-of-service attacks. Furthermore, post-quantum cryptography is becoming a necessity to protect modern critical infrastructure, like high-risk manufacturing, from a potential future quantum-enhanced world. However, lightweight cryptography also maintains sufficient security for applications that do not need quantum-safe security or are much more resource constrained. Both of the cryptographic options may be considered. Additionally, 3D printing security must also consider upholding component and machinery swap capabilities and compatibility with legacy or other devices.

Second, we must minimize the overhead of the security framework to avoid causing significant bottlenecks which affect safety and vital 3D printing components and equipment; while the timing constraints of 3D printers are far less strict than other CAN applications, speed must still be considered to ensure the protocol does not significantly impact operational speed compared to the original, insecure variant. Controller Area Network Flexible Data-Rate (CAN-FD) provides larger, 64-byte payloads at a faster rate and higher packet efficiency compared to CAN, but still requires multiple packets-per-message to send cryptographic materials like public keys and ciphertext. Depending on the environment’s security criticality or resource constraints, the selection of proper encryption schemes can change the number of messages and the amount of overhead imposed on the system by the framework. Thus, minimizing unnecessary packet usage and maximizing the efficiency of messages is also vital in creating incentives for security in devices.

Lastly, security solutions should avoid changes to protocols or damaging compatibility with the hardware and systems already utilized in 3D printing today. Modifying the CAN packet or using sections of the CAN packet for unintended purposes may result in a reduction of the compatibility and feasibility of the system as the CAN back-end and hardware would likely require changes to accommodate for this. Furthermore, changing to a larger, more space-consuming protocol will result in higher costs per part and lessen the incentive for users to adopt security frameworks into their system. One of the most significant benefits of CAN is its ability to maintain a huge number of connections with very few required ports or dedicated hardware components.

Many existing CAN security solutions introduce huge message overhead and/or focus on authenticity checks of all devices within applications which expect few hardware changes, such as within a vehicle’s ECU system [9,10,11]. Some of these frameworks even alter the structure and intention for multiple fields in the CAN packet, resulting in changes to the protocol and possibly the back-end software and hardware that handle such packets [4]. These solutions would thus result in violating the modularity of the system and do not fit the 3D printing system constraints. Similarly to the aforementioned frameworks, our design would require a method of generating secure, consistent private keys, as discussed in Section 4. As mentioned in prior literature, the use of a Physically Unclonable Function—PUF—in this design would provide node authenticity while being a cheaper alternative compared to costly secure key storage, trusted platform modules (TPMs), etc. [12].

Furthermore, many security frameworks for 3D printers apply solutions that require an external device to monitor cyber–physical sensor/device manipulation and command alteration through adversarial attacks, such as machine learning or block-chain applications [13,14]; while these methods offer strong protection, they also require significantly higher computational resources, additional hardware, alterations to protocol, and structural changes, and can be easily manipulated by false sensor readings through man-in-the-middle attacks. This becomes an even larger concern when applying these methods in a printer farm of potentially hundreds of 3D printers with individual security needs. Integration of security within the local communication between printer MCUs would lower the risk of a breach and mitigate the damage that a compromised device could do using man-in-the-middle attacks on itself and neighboring devices. Furthermore, it could be integrated with other work to fully secure the 3D printing pipeline and supply chain by design.

**Figure 2:**
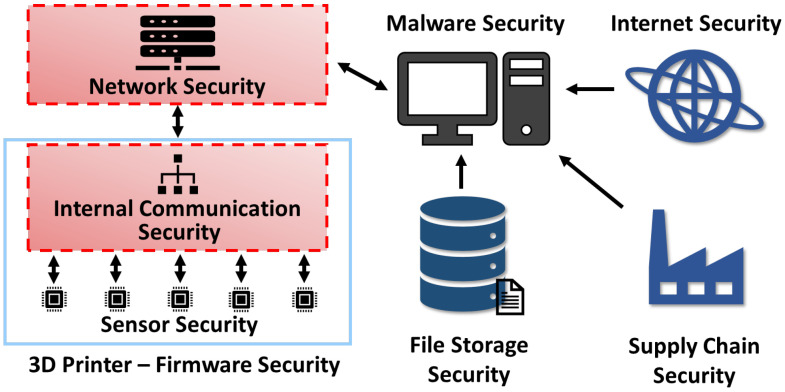
The 3D printing security paradigm and taxonomy of each category of security. Our framework targets the Network Security and Internal Communication Security aspects to secure CAN communications inside and outside the 3D printer and network.

Where this work stands in the 3D printing security paradigm is shown by Figure 2, which demonstrates our system secures two critical structures of the AM taxonomy. As stated before, much existing 3D printer research work focuses on the security of the IoT and controlling server/PC, such as securing against modification using malware or STL file attacks [15,16], supply chain and other related attacks to weaken the printer’s structural integrity [17,18], and attacks on vulnerable firewalls from the greater internet on the local LAN communication [19]. Additionally, some work explores improving the network security of the system using various unique encryption or authentication methods [14,20]. These security systems are discussed further in Section 3. Our design provides novel security in networking to other printers (or devices), and internal communication security using a secured Controller Area Network bus. Little existing work focuses on this important aspect of security in 3D printers despite their huge emphasis on interchangeable parts. Thus, using our design in conjunction with security targeting other taxonomy topics is greatly recommended.

## 3. Background

### 3.1. Prior Related Work in CAN and 3D Printing Security

Security solutions for 3D printing systems have focused on utilizing the power and capability of the controller PC, such as a server desktop, to perform intrusion detection and encryption of GCode or other command and model data. Existing efforts in this direction include digital audio signatures captured from on-site monitoring [17], and machine learning anomaly detection of physical defects and cyberattacks [13,18]. Additionally, encryption, hardware-rooted trust, and embedded watermarking of 3D printed parts have been addressed in prior work [16,20], including the use of block-chain for cryptographic security [14]. An extensive survey was performed that covers existing vulnerabilities and security measures, including the physical communication protocol vulnerabilities we address in this work [19]. However, little work has been conducted to mitigate these vulnerabilities, which demonstrates the novelty of our work in the field of AM security.

Methods of protecting the CAN bus range from server ECU-based authentication of every CAN device to intrusion detection on the CAN bus. ECU authentication methods usually focus on statically defining and storing cryptography or identification material that can provide authenticity, such as digital certificates [11], PUFs [12], firmware digests [21], and co-channel RF watermarking [22]. Furthermore, some work has been done on Post-Quantum Cryptography for CAN encryption in vehicles [11,23]. An in depth survey on intrusion detection using machine learning was previously proposed that studied detection methods for DoS attacks, fuzzing attacks (injection), impersonation attacks, and more [24]; while these methods are powerful detection schemes for vehicles, preventing intrusion and malicious attacks before they can happen would be preferable, especially for alternative CAN bus applications like 3D printing.

### 3.2. 3D Printing (Additive Manufacturing)

**Figure 3:**
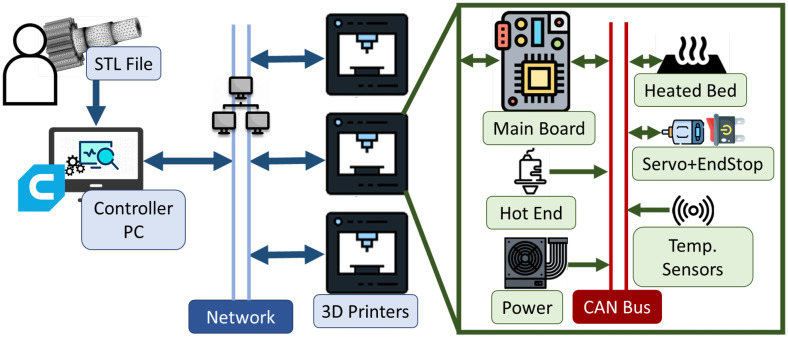
Depiction of an example CAN-based 3D printer structure. The components connect to a CAN bus to be directly addressable by the main board. Each printer in a printer network (or Farm) is connected to the Controller PC through various protocols, the CAN bus being a notable example.

3D printing is the process of applying layers of additive material, such as plastic, to physically create designs specified by GCode/STL files [25]. These printers utilize multiple cyber–physical devices to help in this process, from thermocouples and resistive heaters to servo motors with positional awareness. Marlin [26], a popular open-source framework designed around the RepRap movement, forms the foundation of many commercial 3D printer firmware and software packages. Recently, commercial 3D printing solutions have begun to include one or more IoT-enabled devices within the printers, allowing a PC to control the 3D printer remotely via the internet. Furthermore, 3D printing farms have been popularized in large-scale manufacturing and cloud printing services due to their ability to generate complex structures. These mass arrays of 3D printers all connect to large servers capable of controlling multiple printers at the same time. The CAN protocol has been utilized in previous literature to network 3D printer farms and peripheral devices, like cameras, together in place of an Ethernet LAN [5]. Figure 3 depicts an example of a CAN-based 3D printer structure for both component communications and secure networking to the controller PC.

Prior work in discovering vulnerabilities in popular 3D printers and their common firmware, Marlin, has been an ongoing topic among researchers. Popularity in 3D printer security began with a study focusing on discretely modifying model GCode using malware [15]. This attack raised concerns when they were able to inject a weakness in the 3D-printed drone propeller’s model, causing the part to fail shortly after printing. Furthermore, attacks on 3D printing files and networks have been demonstrated which capture/steal model information and inject subtle, malicious changes to the data to cause printer malfunctions or product failures [27,28,29]. Other attacks focus on using machine learning and other methods to reconstruct GCode by gathering sensor data and monitoring the physical properties of the printer [30]. Lastly, the CAN bus has previously been exploited in commercial 3D printers to modify target values and feedback loops through replay attacks and impersonation. The study demonstrates the malicious modification of target and current temperatures of the printing material and bed, end-stop limit switches, movement axes, and more to create extremely dangerous conditions [3].

### 3.3. Controller Area Network (CAN)

Controller Area Network, or CAN, is a serial communication protocol that allows message transmission between multiple connected nodes without a master controller [31]. CAN eliminates the need for multi-wire loom connections by reducing the communication paths of multiple devices down to a single differential bus. This means devices can connect to the bus and communicate with other nodes using only a single CAN port/IO, as opposed to one port for each device. CAN also provides message arbitration which handles message collisions; while the original CAN protocol supports only up to 8 bytes per CAN message, an upgraded specification of the CAN protocol, Controller Area Network Flexible Data-Rate (CAN-FD), enables faster speeds and up to 64 bytes per message. As a result, this design will feature CAN-FD. A recent survey paper on CAN in 3D printing detailed the different additive manufacturing applications in which the CAN bus has been utilized [1]. Furthermore, commercial printers are beginning to advertise their use of the CAN bus to connect plug-and-play components for its low-cost, highly modular features [32].

Prior work has highlighted the vulnerabilities of CAN security in robotics and vehicular applications, especially with the rise of Internet of Things (IoT) features and devices. As the CAN protocol was not designed for security, many security issues persist that require a higher-level protocol/framework to account for. Because all messages are essentially broadcast on the same communication bus, attackers only need physical access to carry out a wide range of man-in-the-middle attacks, eavesdropping, impersonation, and denial of service [4]. The CAN protocol uses unencrypted messaging with only a destination/ID field for message filtering and arbitration purposes. This restriction causes CAN to lack the ability to authenticate message sources, making all nodes blindly accept any packet that matches their filtered ID (or filter mask). Reconstruction of command structures from eavesdropped CAN packets can be used to perform replay or impersonation attacks as any component on the 3D printer CAN bus. The message collision and arbitration methods of CAN establish a priority system that has been exploited by attackers to perform simple denial of service attacks on CAN buses by constant transmission of high-priority messages [33].

### 3.4. Lightweight and Post-Quantum Cryptography

Lightweight cryptography (LWC) is a design paradigm for cryptosystems which focuses on reducing the required computational power and crypto-material sizes of these systems. One example of such a system is ASCON-128 [34], which is the NIST standard selection for lightweight symmetric encryption for low-resource applications. ASCON-128 also enables the use of nonce values to protect against replay attacks and contains an 8 to 16-byte authentication code, or tag, to ensure message validity. Another LWC system, Four-Q, provides elliptic curve cryptography at the 128-bit level [35]; while we provide both a Lightweight and Post-Quantum implementation of this framework, we highly recommend using the PQC implementation unless resource restrictions require use of the ASCON-based LWC version.

Shor’s Algorithm [6] and Grover’s Algorithm [7] are quantum algorithms which endanger the use of both long-term asymmetric keys and low-security symmetric encryption schemes for critical and high-risk computing and infrastructures. Popular asymmetric encryption schemes, such as Elliptic Curve Diffie–Hellman (ECDH) and RSA, are deemed to be insecure against quantum computation as they become crackable in polynomial time. Applications that utilize these schemes for long-term key establishment are likewise deemed insecure against quantum computation and require new algorithms and cryptosystems to secure them from the potential quantum future. Thankfully, quantum-safe key establishment algorithms have been developed and refined over several years which maintain a high security level against both classical and quantum computing. The NIST has selected the *CRYSTALS-Kyber* post-quantum public key cryptography system for security-critical applications needing key establishment, including manufacturing. Furthermore, classical symmetric schemes (such as AES256) are also considered secure as long as their targeted bit-security level exceeds double the security requirement (i.e., 256 bit security needed to achieve 128-bit quantum security). Similar to ASCON, AES provides the ability to add nonce/counter randomization and authenticated tags with associated data included. As these are computationally expensive, careful consideration is required to efficiently apply them to IoT applications.

## 4. Design of Our Proposed Hierarchical CAN Framework

**Figure 4:**
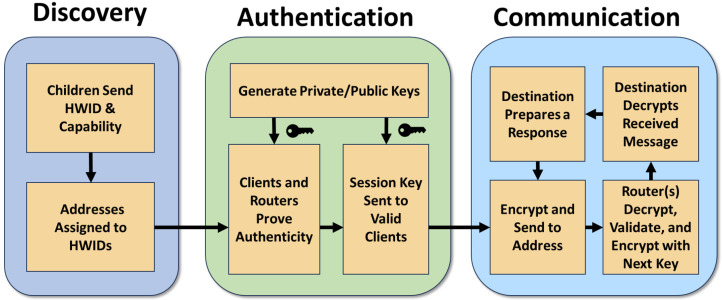
Pipeline design of our framework from initialization to normal operation for a singular subnet. The three distinct phases of our design are discovery, authentication, and communication (including capability and address requesting). Each subnet should perform these steps upon startup.

Our proposed framework for both internal and external/networked 3D printer connectivity utilizes a hierarchical, tree-based design for security with root-of-trust authentication and encryption. The source of trust originates from the controller PC and is negotiated using two-way authentication down each layer/depth of the tree by “routers”, providing a strong, highly secured, and authenticated design for infrastructural devices. We recommend authenticity be provided using Physically Unclonable Functions (PUFs), but the choice is abstracted away in this framework to support any form of secure storage/generation method (i.e., TPM, secure storage); while other communication schemes are compatible with our design, we focus on and recommend the adoption of CAN-FD (which we will generically refer to as CAN) for communication within the 3D printers for its multiplexing broadcast, built-in error, collision correction, and sufficiently-sized payloads. Our design converts a singular CAN network into multiple subnets controlled using “router” devices that handle authentication and message forwarding between the various subnets along the tree’s greater network. Endpoints, or connected 3D printer components, develop individual trust to the router and are isolated in groups to mitigate potential impersonation and eavesdropping of critical components of the bus. The general pipeline design is shown in Figure 4. All three sections of this design are detailed in the following subsections.

Our framework is defined with both lightweight and post-quantum cryptography in mind, with changes to the authentication and encryption structures to support these schemes. As such, our work can support any form of cryptosystem that fits the block size and security requirements for either the pre-quantum lightweight or quantum-safe PQC designs. Our PQC framework requires 256-bit security or higher symmetric encryption with block-sizes smaller than 56 bytes, a quantum-safe asymmetric scheme with at least 128 bits of security, and a hashing algorithm capable of generating at least 256-bit hashes (for 256-bit keys). In this PQC framework, we use AES256-GCM [36], CRYSTALS-Kyber-512 [37], and SHA3-256 for symmetric, asymmetric, and hashing functionalities, respectively; while AES and SHA256 are well known and standardized cryptosystems, CRYSTALS-Kyber is the recently selected NIST standard for post-quantum asymmetric cryptography [38]. Kyber-512 utilizes public keys of 800 bytes, ciphertexts of 768 bytes, and is computationally expensive, requiring our design to carefully consider performance, storage, and message cost constraints. Additionally, AES256-GCM mode is utilized for its counter and authenticity tags. Authenticated encryption with associated data (AEAD) is utilized in both symmetric schemes to ensure credibility of the unencrypted arbitration data of CAN and our framework. These cryptographic systems may be swapped out with other post-quantum algorithms, but careful consideration of security level should be carried out when doing so.

For our lightweight cryptography implementation, we utilize ASCON-128 [34], FourQ ECDH [35], and PHOTON [39] for symmetric, asymmetric, and hashing requirements, respectively. ASCON has been selected as the NIST standard for lightweight cryptography, while PHOTON is one of the standardized lightweight hash-functions from the International Organization for Standardization (ISO) [40]. Unlike PQC, these algorithms utilize very small keys and ciphertexts, with 128-bit (16 byte) encryption keys and 64-bit block sizes (128 bit block size is available). Additionally, ASCON-128 provides authenticity tags and nonces that are used to validate incoming ciphertexts. Similar to the PQC version, any of these can be swapped out for other, similarly sized ciphers.

The proposed framework has three core elements: discovery (and addressing), authentication and cryptographic material establishment, and normal communication. The discovery and authentication will be performed upon every startup of the system, generating new, high-entropy keys for normal communication each time. After startup, address requesting can occur throughout the tree to request capability and address information of all the nodes in the system. This is vital for a system with a non-deterministic structure and interchangeable parts that can have a wide variety of configurations (such as 3D printers with “3-in-1” tool capabilities). The rest of this section describes each of the phases in greater detail.

### 4.1. Discovery and Addressing

As seen in the discovery section of Figure 4, node discovery is the first phase of our design, and the process by which nodes in a given subnet make themselves known and are assigned an address by their parent router. The overall structure of this process is similar to that of the Dynamic Host Configuration Protocol (DHCP), but is initiated through the parent router, rather than through connecting nodes. The discovery process begins with a broadcast by the parent, which prompts all nodes to respond to be assigned an address. The response packet contains the node’s Hardware ID (HID) and other fundamental information, including the primary capability of the node. Because the node has yet to be assigned an address, the nodes respond as the broadcast address, with the HID temporarily serving as a means to differentiate nodes. The parent router then sends a broadcast in response to each node, containing that node’s HID and assigned address. After a configurable wait time (125 ms in the current implementation) the parent node stops listening for discovery responses and begins the authentication process. The protocol was designed with standard CAN hardware in mind, such that devices will always have a CAN filter listening on the broadcast address, and configure an additional filter to listen for their assigned address once they receive one.

**Figure 5:**
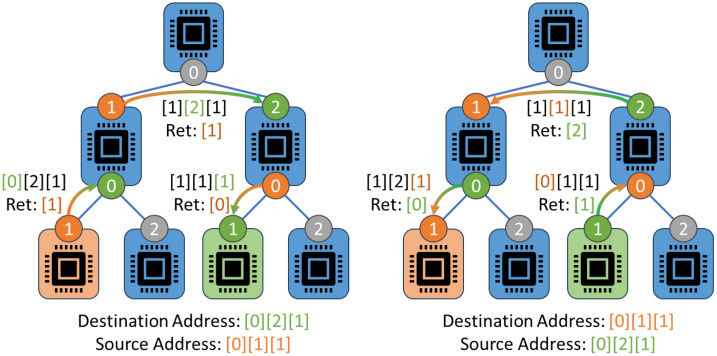
Addresses and routing between a pair of network nodes. The left side shows the route from the orange node (bottom left) to the green node (bottom center), while the right shows the reverse route. Each route is composed from a series of local network address “hops”. The current address is replaced with the return address after each hop, allowing the return route to be calculated.

Our framework uses the 11-bit CAN ID field to perform local addressing within a subnet, alongside a forwarding system which uses variable-length addressing to allow communication between subnets. Local addresses are assigned by the parent router of each subnet during discovery (addresses are sequential in the current implementation). Because the tree structure allows only one route between each pair of nodes, our variable-length addressing scheme uses the intended route of a packet as an address. An example is shown in Figure 5. As shown in the figure, the variable length address is composed of the local address of each intended hop for a given packet. In addition to removing the need for routers to possess complex routing logic, this system also allows the source address for a message to be gradually constructed as the message is forwarded along the tree. After each hop, the portion of the address associated with that hop is overwritten with the return address for that hop. Performing this step after each hop and inverting the hops in the resulting address produces the return address of a given message. Calculating the return address for a message in this manner also makes spoofing a message inherently difficult: even with no additional spoofing protection, nodes can only spoof nodes in their local network, and a router can only spoof parts of a route which precede them. We also implement a Time-To-Live (TTL) field, which serves the dual purpose of preventing infinite circular routing and enabling routers to keep track of the next intended destination of a packet.

### 4.2. Authentication and Cryptography Establishment

Authenticity is a very important factor in the security of this framework’s infrastructure, such as routers and root connections. After initial device discovery and address allocation, the network begins the authentication phase, indicated in Figure 4. In this phase, the trusted PC or micro-controller acts as the controlling—or root—node. This node begins a two-way authentication process with the routers, which then validate their own child subnet (for which they are the server/parent). As this authentication process cascades down the tree (in parallel), each router will authenticate their parent and any routers within their child subnet (two-way auth), ensuring all devices trust their parent and prove trust to their children. Finally, the endpoints will perform validation of their router (one-way auth) and establish secure communication if the router’s authenticity is accepted. Alternatively, endpoints can also be two-way validated in the same scheme as the routers. Trusted or accepted child nodes will receive a randomly-generated session key that will be used in subnet communications and salt the parent session key used for secure forwarding communication. Any node that is not accepted should not receive the session key and should be considered disabled. To minimize timing requirements, the session key can be transmitted safely concatenated to the response hash, as the child device (if applicable) should already be authenticated. Under no circumstances should the shared key be used for normal communication to minimize the risk of key leakage. The authentication scheme for both Public-Key cryptography-based PQC and the Diffie–Hellman-based LWC are provided in the following text. These authentication schemes are similar but utilize different key generation methods.

#### 4.2.1. Lightweight Cryptography Framework

The entire authentication process for the clients (child) and router (parent) when using lightweight cryptography is illustrated in Algorithm 1. The lightweight cryptography scheme utilizes Elliptic Curve Diffie–Hellman (ECDH) to generate shared keys between the devices in Step 1. This means requiring both devices to generate their own respective private keys matching the public keys to ensure the same shared key is created in Step 3, which constitutes the authenticity check of our framework. This approach has been proven to be secure in previous CAN frameworks [12]. In short, the shared secret will be used to generate a temporary secure channel to trade known plain text (or preferably hashed PUF response, if applicable) encrypted by the shared key (Steps 8–19). For optimal security, these shared keys should be hashed afterwards to further obfuscate the original shared secret as seen in Steps 8 and 14. As endpoints do not need authentication in the 1-way method, they may first need to transfer their public key to the server before the shared key can be generated. In Algorithm 1, Steps 7 through 11 occur during two-way authentication, and Steps 12–14 occur during one-way authentication. Note that router authentication (Steps 16–19) needs to be completed regardless in either one-way or two-way authentication. If authentication is successful, a traded random session key is utilized for subnet communication. Secure communication to the router utilizes a unique router session key, $SK_{Router}$, generated through hashing the session key, $K_{Session}$, salted with the shared authentication key, $SSec_{i}$—shown in Step 19 as $SK_{Router}=Hash\left(K_{Session}|SSec_{i}\right)$. All nodes will then communicate using these two session keys for the remainder of the session.
**Algorithm 1** Lightweight Cryptographic Authentication (1-way and 2-way)  1:**Input: **Clients** Output: **SessionKeys,ValidNodes  2:Generate private key **$S_{C}$** and private key **$S_{R}$** for Clients and Router, respectively.  3:Router hashes private key to generate challenge hash $H_{R}$.  4:Router generates a random Session Key, $K_{Session}$.  5:Clients use stored Router Public Key $P_{R}$ and Router uses Clients’ Public Keys $P_{C}$.  6:**for **i←1,numClients** do**  7:    $\mathrm{Client}\leftarrow Clients_{i}$  8:    **if** Client is also a Router (Two-Way Auth) **then**     ▹ Client Authentication  9:        Generate and hash ECDH shared key SSec for Client ($S_{C_{i}}$, $P_{R}$), Router ($S_{R}$, $P_{C_{i}}$).10:        Client hashes private key to generate challenge hash $H_{C_{i}}$.11:        Client sends encrypted $H_{C_{i}}$ to Router using $SSec_{i}$ shared key.12:        Router decrypts $H_{C_{i}}$ using $SSec_{i}$ and validates it to stored authentic hash.13:    **else if** Client is an Endpoint (One-Way Auth) **then**   ▹ **No** Client Authentication14:        Client transmits their Public Key $P_{C_{i}}$ to Router, if necessary.15:        Generate and hash ECDH shared key SSec for Client ($S_{C_{i}}$, $P_{R}$), Router ($S_{R}$, $P_{C_{i}}$).16:    **end if**17:    Router sends encrypted $H_{R}$ to Client using $SSec_{i}$.     ▹ Router Authentication18:    Client decrypts $H_{R}$ using $SSec_{i}$ and validates it to stored authentic hash.19:    Router sends encrypted Session Key $K_{Session}$ to Client using $SSec_{i}$ shared key.20:    Both generate Router Session Key by $SK_{Router}\leftarrow Hash\left(K_{Session}|SSec_{i}\right)$.21:**end for**22:Router signals to all Clients that authentication is complete.23:**Return** Session Keys, Valid Nodes

#### 4.2.2. Post-Quantum Cryptography Framework

The post-quantum (PQC) scheme features higher security, a different style of asymmetric cryptography, and higher computational and message costs. The PQC authentication steps are illustrated in Algorithm 2. This scheme uses public-key cryptography (PKC) to encrypt shared secret material with public keys and generate a shared AES-256 key of 32 bytes. To begin, after key initialization (Steps 1–4), an additional initial step of transmitting the client’s public key (Step 7) can be utilized to save on precious embedded resources on the chip. This public key can be hashed and compared to a stored, smaller 32-byte hash stored in the parent/server for the two-way (or router) authentication scheme. Alternatively, these public keys can be stored within each system to reduce the number of messages required. Similarly to LWC, the public key transfer in Step 7 is non-optional for one-way endpoint authentication as the endpoint’s public key is not known to the server. In the two-way authentication scheme, both the client and router will encapsulate a random 32-byte shared value with Kyber using the other’s public key and trade the ciphertext (Steps 8–18). Next, they decrypt the information and XOR the two generated keys together to generate a combined authentication key (Step 16). In order for this secure channel to work, both sides must be authentic (the owners of the respective stored public keys) to ensure that the XOR produces the correct value, providing a strong authenticity check. Using this secure channel, they will then trade known ciphertexts, such as PUF response hashes, before the server provides the 32-byte randomly generated session key sequence to the client (Steps 25–28). In one-way authentication (shown in Steps 19–23), only the client will transfer a 32-byte challenge ciphertext and both parties will use this for the shared key authentication (Step 23). In both schemes, the client will utilize the session key, $K_{Session}$, for CAN inter-network communication. Similarly, a unique router session key, $SK_{Router}$, is also generated using a hashed $K_{Session}$ salted with the shared key $SSec_{i}$ by $SK_{Router}=Hash\left(K_{Session}|SSec_{i}\right)$ (Step 28).
**Algorithm 2** Post-quantum cryptographic authentication (1-way and 2-way)  1:**Input: **Clients** Output: **SessionKeys,ValidNodes  2:Generate private key **$S_{C}$** and private key **$S_{R}$** for Clients and Router, respectively.  3:Router hashes private key to generate challenge hash $H_{R}$.  4:Router generates a random Session Key, $K_{Session}$.  5:Clients use stored Router Public Key $P_{R}$ and Router uses Clients’ Public Keys $P_{C}$.  6:**for **i←1,numClients** do**  7:    $\mathrm{Client}\leftarrow Clients_{i}$  8:    Client transmits their Public Key $P_{C_{i}}$ to Router, if necessary.  9:    **if** Client is also a Router (Two-Way Auth) **then**     ▹ Both Authenticated10:        Router should validate hash of transmitted $P_{C_{i}}$ with stored hash, if applicable.11:        Client hashes private key to generate challenge hash $H_{C_{i}}$.12:        Client generates random 32-byte key $Ch_{C}$ and encrypts using public key $P_{R}$.13:        Client transmits Kyber-encrypted $Ch_{C}$ to Router.14:        Router generates random 32-byte key $Ch_{R}$ and encrypts using public key $P_{C_{i}}$.15:        Router transmits Kyber-encrypted $Ch_{R}$ to Client.16:        Router decrypts $Ch_{C}$ using $S_{R}$ and Client decrypts $Ch_{R}$ using $S_{C_{i}}$.17:        Both combine keys to make Shared Key: $SSec_{i}\leftarrow \left(Ch_{C}⊕Ch_{R}\right)$.18:        Client sends encrypted $H_{C_{i}}$ to Client using $SSec_{i}$.19:        Router decrypts $H_{C_{i}}$ using $SSec_{i}$ and validates it to stored authentic hash.20:    **else if** Client is an Endpoint (One-Way Auth) **then**  ▹ **Only** Router Authentication21:        Client generates random 32-byte key $Ch_{C}$ and encrypts using public key $P_{R}$.22:        Client transmits Kyber-encrypted $Ch_{C}$ to Router.23:        Router decrypts $Ch_{C}$ using $S_{R}$.24:        Router uses challenge as Shared Key: $SSec_{i}\leftarrow Ch_{C}$.25:    **end if**26:    Router sends encrypted $H_{R}$ to Client using $SSec_{i}$.27:    Client decrypts $H_{R}$ using $SSec_{i}$ and validates it to stored authentic hash.28:    Router sends encrypted Session Key $K_{Session}$ to Client using $SSec_{i}$ shared key.29:    Both generate Router Session Key by $SK_{Router}\leftarrow Hash\left(K_{Session}|SSec_{i}\right)$.30:**end for**31:Router signals to all Clients that authentication is complete.32:**Return** Session Keys, Valid Nodes

### 4.3. Normal Communication

**Figure 6:**
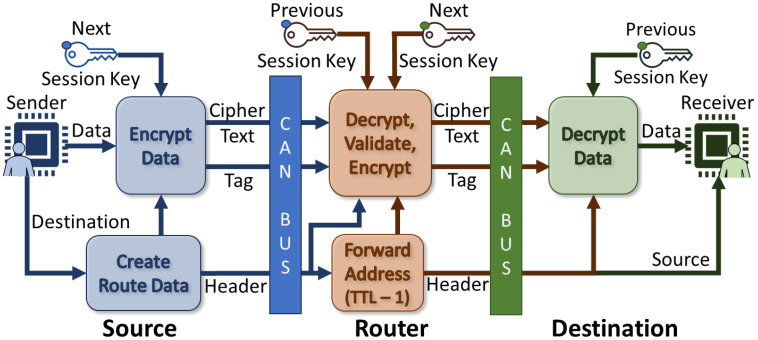
General flow of standard communication depicting a sender transmitting through a router to reach a destination. Source (blue) creates the route and encrypted data to send to the first “hop” in the series. The router(s) (orange) will decrypt with the previous bus’ session key, validate, and re-encrypt the data using the next unique CAN bus parent session key. The router process is repeated until the data reaches the destination (green). Addresses and TTL are modified after each hop.

The final phase of our design is normal communication, represented as a loop in Figure 4. When any device wishes to connect to a node outside of its own CAN subnet, it will first ensure they have acquired the address of the destination from an authenticated authority node, such as requesting the root. The device will then need to fill in the appropriate address, length/TTL, data, counter/nonce, etc. All packets’ data fields must be encrypted (unless defined otherwise by the application) using the most up-to-date pair of session keys established in the authentication phase, including the tag for authenticity checks. For communications to routers, devices must encrypt using the router session key generated previously for secure router-to-endpoint communication. Otherwise, local subnet communication can be encrypted with the subnet session key to talk to neighbors without the need for router forwarding. When encrypting packets before transmission, associated data (AD) or similar features (authenticated encryption with associated data—AEAD) should be utilized to prevent the CAN headers and our framework headers from being modified, as they cannot be encrypted. Routers will forward the packet information based on the address and TTL fields to the correct location, destroying/stopping any packet that does not pass the encryption and authenticity check and application-designed firewalls (if any). This general flow from sender to receiver can be seen in Figure 6.

**Figure 7:**
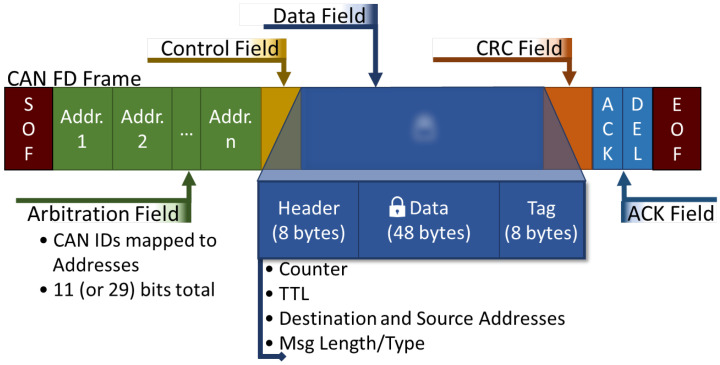
The Controller Area Network Flexible Data-Rate (CAN-FD) frame with the data field divided into sections required for our framework. Use of a header of at least 8-bytes is recommended, leaving the data and tag fields with 56 bytes to use for encrypted data.

In normal communication, all messages are encrypted using the run-time session keys generated previously in authentication. This encryption utilizes all the additional security features included with the chosen symmetric encryption scheme, including authenticity tags to prevent fuzzing/impersonation and counters to prevent replay attacks. The typical CAN-FD payload offers a 64-byte data field to operate with. In our normal communication, eight of those bytes are reserved for the routing system to allow for header information (counter, address, TTL, etc.) without compromising the CAN-FD protocol itself. That allows for 56 bytes to be utilized for the data, including the tag (which is usually 8–16 bytes). A figure detailing a CAN packet containing all these features is shown in Figure 7; while our protocol takes up some of the CAN-FD data field, it remains still faster and more efficient than the 8-byte fields of CAN and is still efficient enough to maintain 3D printing payload and performance requirements as shown in Section 6.

In addition to basic communication, our framework provides a few simple functions to components connected to the network, most notably the ability to request addresses or capabilities of network nodes. Each node is associated with a capability which describes the function of the device; examples include servo, temperature sensing, and fan capabilities. We include a command for requesting the network address of nodes with a given capability along a subnet, which allows, for example, the address of a temperature controller on a subnet to be retrieved so that it may be queried for temperature data. We also include a command to request the capability of a node at a particular network address, which enables inspection of the network by routers or the root node.

## 5. Analysis and Discussion of Proposed Framework

In this section, we discuss the security, implications, and design justifications of our proposed design, as well as compare our design to existing methodologies in both CAN and 3D printing. By implementing a hierarchical authentication structure instead of the typical CAN bus, we can protect against attacks that even many popular existing frameworks remain vulnerable to. One such example is denial-of-service mitigation, as minimizing the number of devices dependent on the disabled CAN bus allows most of the tree network to continue normal operation during attacks. Furthermore, our root-of-trust security scheme for both crypto-suites provides robust, secure communication and trustworthy infrastructure to both 3D printing networks and components. As communication keys are ephemeral, compromising any node in the system would require physical access to the secure private key storage/generator (i.e., PUF) to retrieve the means to authenticate and generate the appropriate keys. These private keys should be retrieved from secure storage or generated at run-time as needed and should not be cached or stored in insecure non-volatile memory to avoid key leakage. As public keys are non-sensitive information, they do not need to be kept secret and can be stored within unsecured non-volatile memory, such as flash memory. Endpoint nodes’ public keys do not require permanent storage as these devices can be swapped among routers and are run-time dependent. As hashes are considered irreversible operations, these too can be stored in insecure memory as adversarial theft of hashes would not compromise the system. Furthermore, use of the long-term keys, such as LWC’s shared secret or PQC’s private/public key, should be limited to authentication and ephemeral key transfer only. All encrypted communication after authentication should utilize the temporary session keys generated. Re-authentication of nodes based on a message count or timing system can be configured to provide additional protection against attacks on session keys. This would also give the system the ability to identify devices which have been tampered with or disabled during real-time operation. This process does not require the devices to regenerate and transmit public keys or shared keys. If new routers are added to the system, devices that authenticate them (their parent and children) will require re-enrollment of their public keys, hash, and other necessary data.

**Figure 8:**
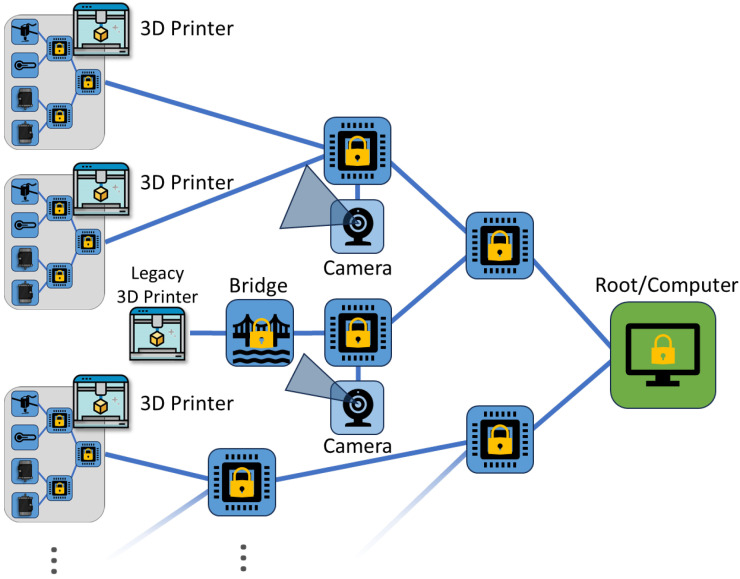
Depiction of the expansiveness of our framework for both internal 3D printer communication and CAN networking. The tree structure allows for printer trees to expand into network trees, as well as allow legacy, non-CAN printers and peripherals (cameras) onto the network safely.

Because we utilize a secure tree structure with maximum modularity, our framework is essentially infinitely expandable. This framework can also be utilized in both the internal CAN connections and expand to connect external, networked CAN 3D printers, as demonstrated in Figure 8. Due to the design of the framework’s header, the tree structure we utilize does not have to be balanced, nor do endpoints have to always be at the bottom level of the tree (although, we recommend only two-way authenticated endpoints for this to minimize the damage of endpoints performing DoS attacks on the subnet routers). This design means that even legacy 3D printers, with or without the CAN bus, can also be connected to the CAN network using a compatible CAN device to bridge the connections and protocol. Our design is also compatible with Marlin-based firmware. The depth and width of the tree and its branches/subnets are heavily dependent on the parameter of max devices allowed in a subnet, or *k*. If *k* is too small, then the network could suffer increased latency due to the increased tree depth required to connect a given number of devices. However, a larger value of *k* will increase the impact an attacker can have on the system, as more devices will be located in the same subnet as the attacker. As shown later in Section 6, the relationship between the number of endpoint devices *n* and the selected parameter *k* is logarithmic in nature.

Additionally, CAN-based 3D printers, such as Snapmaker [32], already utilize a discovery and capability phase in their device startup, making our design fit existing CAN-based 3D printing. However, our goal is also to encourage further use of CAN/CAN-FD in current and future 3D printing devices and applications to improve their design and capabilities. Thus, providing a security solution like this is one of the first steps in promoting such communication design choices.

### 5.1. Normal Communication and Routing

Routers in normal communication are tasked with routing packets between the CAN buses above (parent subnet) and below (child subnet) them appropriately after checking for authenticity and valid routing. Based on typical 3D printer communications, most communication in this stage will be local subnet communication, broadcast commands (i.e., GCode or requests) sent by the root, and query responses for physical sensor/status readings. By routing this way, we also substantially reduce the load on any singular CAN bus in systems with high node counts, as more information is being broadcast down or is isolated in respective subnets. When routers perform forwarding/hopping, each subnet’s encryption key will be used on each hop (decrypt using previous key, encrypt using next key) to ensure that new keys or existing keys do not have to be exposed outside of subnets, strengthening the security of the system and minimizing the number of devices that contain these secure keys. As this is a low-level framework, user applications have the ability to also include an additional layer of protocol to create end-to-end encryption between the devices, such as an SSL-like protocol.

### 5.2. Lightweight Cryptographic Schemes

Lightweight cryptography addresses performance and resource requirements of standard and post-quantum cryptography by providing low-computation and small data security while maintaining an acceptable level of quantum-unsafe security. We utilize standards chosen by both the International Organization for Standardization (ISO) and National Institute of Standards and Technology (NIST) for our lightweight cryptography to inspire high confidence in our security for resource constrained environments. These schemes reduce the computational costs of encryption, key generation, and more for the end user while maintaining acceptable levels of pre-quantum security. Frequently refreshing session keys with re-authentication can greatly improve the security against threats like quantum computers as session keys are completely random (preferably with the highest entropy available).

Systems that utilize this LWC framework should only be systems with low chance of targeting by adversarial actors, such as consumer-level 3D printers or low-significance manufacturing. Additionally, systems which possess extremely low resources or require extremely high performance (and cannot support PQC hardware acceleration) benefit from at least implementing the quantum unsafe scheme; while we recommend utilizing the PQC framework, utilizing classical pre-quantum cryptographic standards as of today remains a robust choice for securing the CAN bus.

#### 5.2.1. Asymmetric Key Generation

We utilize Elliptic Curve Diffie–Hellman (ECDH) based on the FourQ library [35] for key exchange. Any popular security curves, such as Curve25519 from NIST, could work for this task. However, FourQ has been shown to be faster than other 128-bit security elliptic curves [35,41].

#### 5.2.2. Authenticated Encryption and Decryption

For this framework, we selected ASCON-128 bit symmetric encryption due to its selection as the NIST standard for lightweight encryption [34]. The symmetric scheme should utilize block sizes that fit properly within the data field of our CAN system, thus allowing either ASCON-128 (64-bit blocks) and ASCON-128a (128-bit blocks) to be used. Additionally, ASCON provides a 128-bit tag, nonce, and associated data for further replay and impersonation attack protection.

#### 5.2.3. Hashing

We chose the ISO-standardized PHOTON-128 [39] hashing scheme, as the hashing scheme must provide 128-bit hashes to be able to be used immediately as encryption keys. This also makes the hash size smaller than a single CAN frame and a multiple of our symmetric encryption block size.

### 5.3. Post-Quantum Cryptographic Schemes

The performance impact of a pure-software PQC implementation may encourage users to find faster implementations, such as hardware acceleration which can occur in parallel alongside the initialization process. Many TPM modules are beginning to include post-quantum cryptography, including CRYSTALS-Kyber, in their hardware acceleration designs, allowing users to have both a secure method of storing/generating private keys and of generating the necessary public-key cryptography in one package [42]. This would allows users to continue using low-cost micro-controllers while having the benefits of PQC. However, many low-cost, highly optimized implementations of CRYSTALS-Kyber have been proposed that could also be implemented alongside the PUF to provide similar benefits [43,44]. Otherwise, as shown in Section 6, the pure-software implementation of the cryptography still provides high performance in normal communication and acceptable speeds in authentication, especially given that initialization steps have less constrained timing requirements.

#### 5.3.1. Asymmetric Key Generation

For public key cryptography in our PQC design, we chose the NIST standard CRYSTALS-Kyber [37] which targets a 128-bit post-quantum security level for public key cryptography. This provides excellent security against classical computing as well. As this asymmetric scheme is different than Diffie–Hellman, the PQC framework was modified to accommodate the differences.

#### 5.3.2. Authenticated Encryption and Decryption

As quantum experts indicate that the security level of all symmetric ciphers are essentially “halved” against quantum computers, we chose the standardized AES-256 cipher to match the security level of Kyber. However, any cipher with 256-bit security and 16-byte block sizes is acceptable. We choose to use the Galois/Counter mode (GCM) to provide replay attack and message authenticity protection. By utilizing associated data (AD), we can include the unencrypted header fields in the authentication to prevent tempering.

#### 5.3.3. Hashing

Our hashing function should have at least 256-bits to ensure hashed keys have 256-bit security with AES. Comparison hashes, such as the private key and public key hashes, can be truncated to 128-bit to save memory. This also allows for the transferred challenges to be exactly 16-bytes large to match the AES block size. We chose SHA256 for this task.

### 5.4. Threat Mitigation

One of our framework’s primary capabilities is protection against eavesdropped and fabricated messages sent to authority nodes, such as the root device. In standard CAN configurations, an attacker could easily fabricate messages to any destination, including impersonating other devices. The main attack surface our design’s security protects is the bottom-most endpoint (or 3D printing component) layer as many components sourced from potentially insecure supply chains are often swapped around and replaced. We do not discuss concerns of the root (or controller) being compromised as this is outside the scope of this work and has already been addressed in a significant amount of the literature.

Our framework provides source and destination authentication using unique keys, challenge–response authentication upon startup, and counter/tag cryptographic systems. This allows us to prove the validity of and prevent impersonation for all messages being sent in the system. Because the data are in a secure, encrypted state with uniquely modified session keys, eavesdropping attacks are counteracted. The only information an attacker is capable of retrieving is metadata on the CAN header (i.e., destination ID) or framework header (TTL, Addr, etc.), which are all unencrypted for routing purposes. The generated tag (and associated data) secures our design from fuzzing attacks and packet manipulation attacks as secured MACs are calculated and transmitted with every packet to validate both the encrypted and header information. Replay attacks on the authentication phase would immediately be stopped by the randomness in challenge keys and session keys of the frameworks, preventing nodes from impersonating devices during startup. In the case of transmitted public keys, these public keys are hashed and compared to stored registered device hashes. If manipulation occurs during discovery or other initialization phases, it will only result in a denial-of-service behavior that will impact only a single subnet, creating a similar scenario to the CAN subnet simply being disabled.

If re-authentication is utilized, session keys in both of the presented cryptographic schemes change rapidly enough to significantly reduce the impact of even cracked session keys. This does not eliminate the threat of eventual eavesdropping (as past messages can be decoded at anytime if a session key is cracked), it does reduce the impact of any individual key being compromised. Because the session keys are generated and transferred randomly during run time, our design provides forward secrecy (FS). Even if the private key is eventually compromised, the attacker would still be required to brute-force the session key of past communications. If the user requires it, endpoints can easily be reconfigured to perform two-way authentication with the router, requiring the endpoint to be statically defined in the router’s known-authentic device table.

By having routers perform the routing of the packets, a firewall is inherently provided that blocks impersonated traffic, malformed packets, and unauthorized routing. This also means that malicious nodes cannot read traffic coming from other subnets, preventing them from extracting any information. We believe these benefits to greatly outweigh the message/traffic cost of having to redeploy messages along the bus. Denial-of-service attacks cannot breach the inherent rate-limiting and authenticity checks of the router, thus protecting our system from network-wide denial-of-service. As such, attackers are limited to a potential attack surface of only *k* subnet nodes, which our additional security measures protect, authenticate, and secure.

### 5.5. Comparison to Existing Security Frameworks

Our framework has a number of unique features compared to existing 3D printing and CAN security frameworks. However, we can still provide relevant points of comparison between this work and existing work to determine where our work stands in the 3D printing security and CAN security environments. The security holes which exist in these systems have produced a variety of security solutions in either domain. No single solution is able to cover all aspects of the additive manufacturing supply chain and devices. This section will compare our solution to both existing CAN frameworks (including Vehicular ones) and existing 3D printing security schemes. These comparisons are shown in Table 1 and Table 2.

Our design uniquely reshapes the CAN bus in a way that allows CAN devices to have safe, confidential transmissions to and from the central device. Existing frameworks were usually designed with different constraints (vehicular) than our work, resulting in features unsuitable for our application or a lack of vital characteristics required for 3D printing. However, many aspects of our work and existing CAN systems are comparable, including authentication and communication/security along the bus. CAN frameworks are usually structured as a singular bus which contains a server that performs the authentication of nodes, similar to ours. Two-way authentication is the only allowable option for other CAN frameworks. In our design, we must accept 3D printers into the system which could potentially have hardware Trojans and mitigate their damage. Most designs, including this work, utilize secure private key storage or generation, such as PUFs, in place of hard-coded values or other methods. The impact of eavesdropping on our system is much greater than others due to sensitive intellectual property (IP) being transferred, requiring the implementation of additional security concepts like forward secrecy. Message authenticity, fuzzing defense, impersonation defense, and replay defense are as important in our framework as other CAN systems. Our framework utilizes counters and tags to ensure received messages are validated before being processed to prevent fuzzing, replay, and other forms of bus manipulation. Frameworks lacking these features are vulnerable to identical and random ciphertext attacks. Finally, our design maintains a degree of denial-of-service protection, which was an important goal for our design. The tree-shaped structure of our framework prevents attackers from disabling other subnets, mitigating the much of the damage other frameworks would be vulnerable to. These CAN comparisons are shown in Table 1.

Similarly, some comparisons of the general impact and targeted security features of 3D printing security designs can also be qualitatively performed to get a better picture of where our work stands. No solution will be able to provide complete security (including ones not mentioned in Table 2), making it important that these solutions be combined to provide more comprehensive coverage. As our work targets a domain others have not investigated deeply (communication), our work defends against some adversarial threats that fall out of scope for (or are not considered by) other security solutions. Our design actively protects the communications system from tampering, eavesdropping, and impersonation as described previously. As our work is directly located within the 3D printer’s systems, it does not require external computers or servers to perform monitoring of audio, print abnormalities, etc. or host machine learning applications. However, this means that while our system can detect and prevent most attacks entirely, any other forms of attack will not be discovered by our system (alongside some others) and would require intrusion detection models like Beckwith et al. [13] or Belikovsky et al. [17]. These comparisons are shown in Table 2.

## 6. Experimentation Results

**Figure 9:**
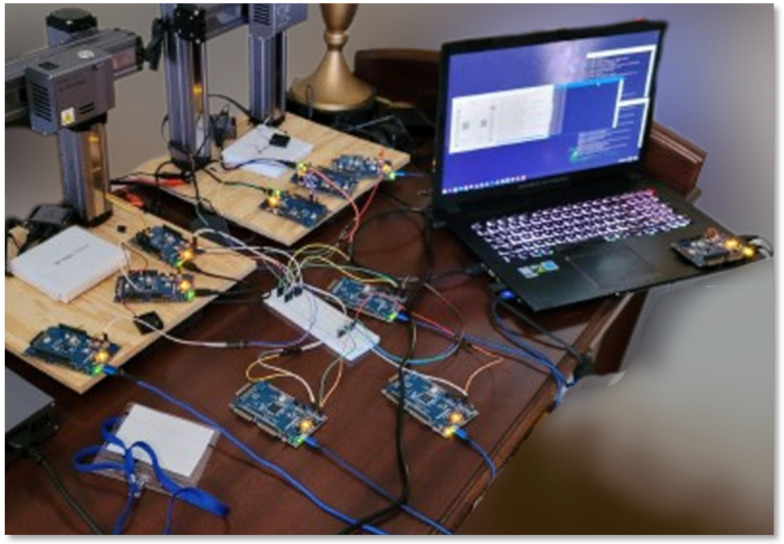
Full test bed of our framework implementation, with attached 3D printer components. Network nodes are implemented by SAM C21 micro-controllers with servo, temperature sensor, and fan components attached to the endpoints.

**Figure 10:**
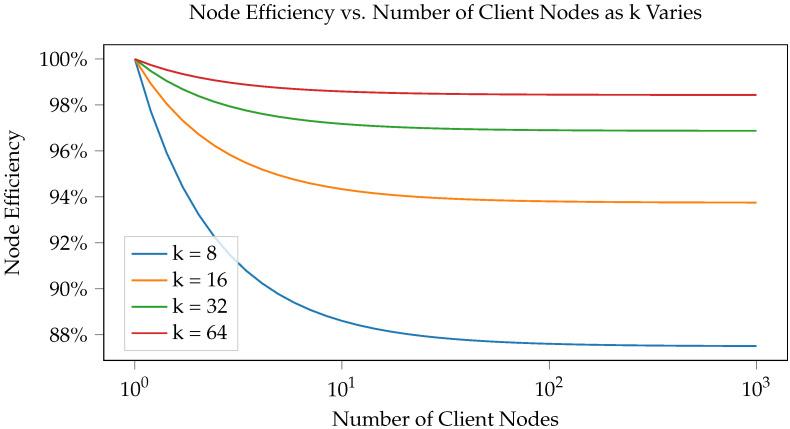
Node efficiency vs. number of client nodes for several values of *k* (number of nodes in subnet). A lower node efficiency percentage means more routers and infrastructure. This directly impacts number of hops (latency), number of branches, and hardware cost of our framework.

**Figure 11:**
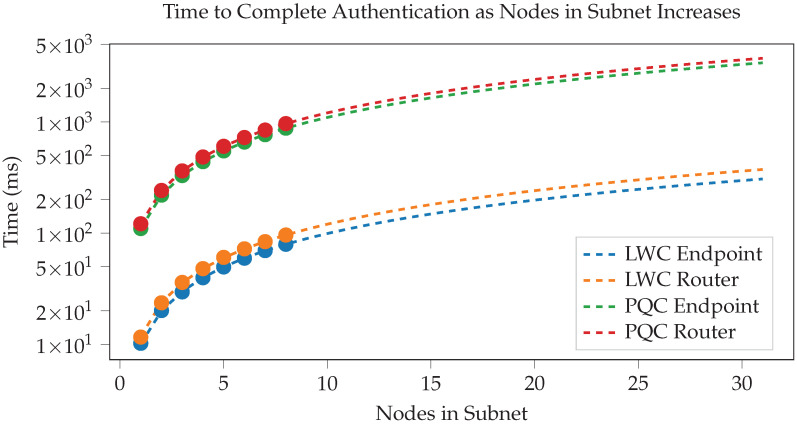
Results of time to complete authentication, in Log(y), for the number of nodes in subnet *k*. Data points extrapolated by line of best fit for high values of *k*. Post-quantum performance gives a 10× overhead, but both are well within acceptable timing ranges for expected values of *k*.

**Figure 12:**
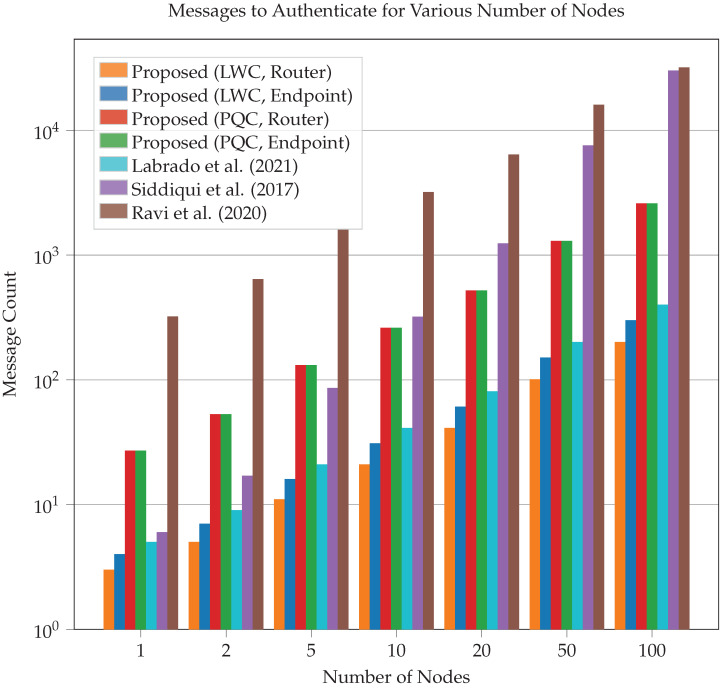
Number of messages (cost) needed to perform authentication in our LWC and PQC frameworks compared to existing CAN frameworks. Our LWC and PQC frameworks have noticeably different message costs, but both still perform better than most other CAN frameworks [10,11,12].

**Figure 13:**
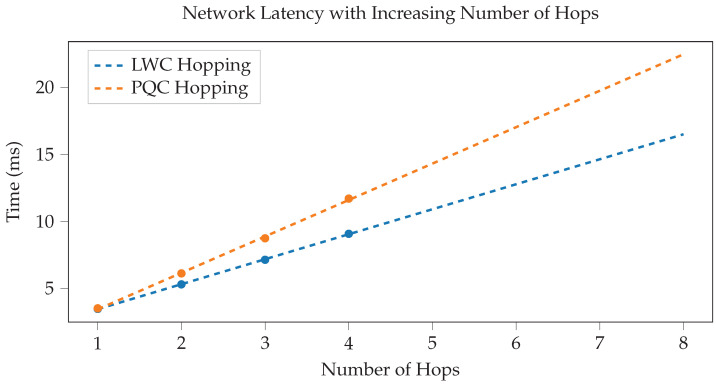
Network latency (time taken for transmission) for lightweight and post-quantum framework designs by number of hops. Each hop requires a router to validate and reconstruct each packet. Maximum number of hops is usually limited to two times the depth of the tree.

To draw conclusions about the performance of our framework, we created a working, generalized implementation of our design on a CAN-FD network composed of Microchip SAM C Arm Cortex-M0+ based micro-controllers. These ARM-based MCUs have a clock frequency of 80 MHz, 256 KB of flash, and 32 KB of RAM. An example configuration of this implementation is shown in Figure 9. To ensure that both encryption suites can be accurately compared, we utilized pure-C (software-only) implementations of official sources for every cryptographic algorithm. Thus, these results demonstrate that the baseline (possibly worst-case scenario) performance is still very fast and sufficient for 3D printing. This also means that cryptographic results can be greatly improved by hardware acceleration and MCU-based optimizations. The tests we performed include the hardware efficiency cost of our system based on chosen *k* (Figure 10), the time taken to authenticate a subnet based on *k* (Figure 11), the CAN message cost for authentication compared to other frameworks (Figure 12), and network latency for normal communication based on hops (Figure 13). This section will discuss these implementation test results.

### 6.1. Hardware Efficiency Cost

One major constraint of our design is the requirement of additional micro-controller units for tree infrastructure, such as routers and the root. The structural constraints of the tree and the total number of endpoints/clients needing to be connected both affect the number of routers required. We define a useful metric, “node hardware efficiency”, to evaluate the effect that *k* has on hardware cost for various numbers of client nodes. Hardware efficiency can be defined as $Efficiency=NumNodes_{Endpoint}/TotalNodes$, meaning a higher percentage indicates less infrastructure is needed to design the tree. For example, 100% efficiency means that no additional hardware overhead is needed to utilize our framework.

This relationship is demonstrated in Figure 10, where the node efficiency is compared to the number of client nodes based on subnet *k* sizes of 8, 16, 32, and 64. A logarithmic relationship can be seen between node efficiency and the number of client nodes, with the size of *k* altering how much node efficiency degrades before leveling out. Higher values of *k* improve the node efficiency for any number of nodes, resulting in approximately 99% node efficiency. As an example, when the value of k=8, our system has at least 88% node efficiency. This means that at most 12% more hardware is required to use our approach. This graph allows us to make several relevant conclusions. First, we know that larger values of *k* mean the tree scales more efficiently, requiring fewer routers to handle a given number of devices. This means the infrastructure requires fewer changes in structure to introduce a large number of nodes. Second, the node efficiency is indirectly correlated with the depth of the tree, and as a result, it is correlated with the maximum number of hops required for messages. Lower values of *k* will result in the messages needing to pass through more routers and infrastructure to reach their destination. Finally, as *k* increases, the attack surface for an adversarial hardware Trojan increases because more devices utilize each CAN bus. Thus, we can reasonably conclude that the adversarial impact is negatively correlated with the node efficiency, producing a trade-off between performance and security (as expected). Thus, we recommend selecting moderate values of *k* to avoid an extreme security and hardware cost trade-off, such as k=16.

### 6.2. Authentication

The authentication process which is performed on initialization is vital to the security of our design. This step provides the root-of-trust and establishes all the cryptographic keys and structures that will be used for the entire rest of the session. Thus, providing sufficient security to prevent attackers from being incorrectly validated as critical infrastructure was the core focus of our design. Since this stage is performed while the system is relatively inactive (during initialization) performance is not a crucial metric. However, a strong CAN framework should still consider optimization, message cost, and total run time to prevent authentication (and especially re-authentication) from bottlenecking the system, blocking critical safety messages, and/or removing incentive for users to adopt the framework.

Our framework attempts to minimize the numbers of transmissions that are required for authentication. To demonstrate how strong our authentication scheme is, we have compared the number of messages required to that of existing CAN security frameworks to get a general idea of how efficient each framework is. The number of messages for each value of *k* CAN bus nodes is located in Figure 12. We can see that our lightweight cryptographic framework performs best out of the frameworks given in the graph, with about a 25% decrease in packets required from an existing lightweight CAN framework, Labrado et al. [12]. Furthermore, our PQC framework performs better than existing CAN post-quantum frameworks, such as Ravi et al. [11], requiring approximately 90% less packets. All four frameworks also outperform non-linear scaling frameworks, with the performance improvement increasing for higher node counts. Our results include the public key transfer in PQC mode for both routers and endpoints, resulting in equal message costs.

Authentication is separated into either two-way or one-way validation, as described in previous sections. We gathered timing results for both endpoints (one-way) and routers (two-way) in both post-quantum and lightweight cryptography methods. The results can be seen in Figure 11, with trend lines extrapolated from experimentally collected data. It should be noted that the y-axis is logarithmic to allow for better comparison of LWC and PQC results. Our graph is extrapolated for values of *k* from 9 to 32 to see the trend for larger subnets. As expected, the time taken for our authentication scheme is linear with little variation, increasing as the subnet size increases. For recommended values of *k*, we see acceptable run times for authentication, especially given that authentication occurs during startup (and thus likely before critical communications are necessary). All cryptography is in pure-C implementation without optimization or hardware acceleration. This means that while the linear behavior will be consistent, these timing results could be “shifted” down the y-axis significantly by applying hardware acceleration and assembly (or other) optimization.

### 6.3. Normal Communication Latency

**Figure 14:**
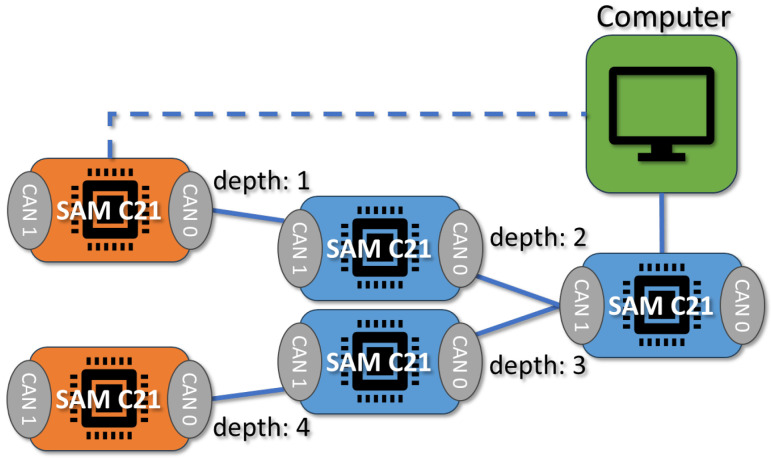
Network configuration for measuring normal communication latency. The SAM C21 micro-controllers are connected in our tree-based network, with the computer connecting to both the root (solid line) and one endpoint node (dashed line). Latency tests are conducted between each message hop count (or depth) and extrapolated, as shown in Figure 13.

After authentication, normal operation involves transmitting messages along the CAN buses, either between subnet devices or through routers to a verified destination. Routers are required to decrypt, validate, and re-encrypt packets traveling between subnets to ensure that the traffic is secure and authentic. Because packets must been decrypted and re-encrypted once per hop, the performance of the symmetric encryption algorithm is vital to the performance of the framework. To measure network latency, we constructed a test setup which allows for a ping test to be sent across varying number of hops, shown in Figure 14. Results showing the network latency (or performance) of our framework based on the increasing number of “hops” through routers is shown in Figure 13.

We can see that hops follows a linear trend similar to authentication, increasing approximately by 3 ms for each hop in post-quantum, and 2 ms per hop in lightweight cryptography. The maximum number of hops required to reach a destination scales with the depth of the system, which is in turn influenced by the subnet size, *k*, and other variables. For example, a message from an endpoint node would need to hop up the full depth of the tree to reach the root node. Our results indicate that even relatively high hop counts reach only approximately 20 ms and 15 ms for PQC and LWC, respectively. This too is a figure that can be reduced by applying hardware acceleration, optimization, and other algorithm performance improvements. However, regardless of algorithmic optimizations, the performance will still follow a linear trend.

## 7. Discussion and Concluding Remarks

In this paper, we present a novel security framework for 3D printing (additive manufacturing) based on a hierarchical CAN bus structure built from root-of-trust. The proposed framework offers 3D printing applications strong security that takes plug-and-play and post-quantum protection into consideration; while many 3D printing security solutions exist, our work is one of the first to address security in the internal communication systems (i.e., CAN). Our design addresses security concerns for untrusted 3D printer components that are regularly swapped, such as hardware Trojans. Our framework provides improvements compared to existing CAN frameworks for other applications and brings new ideas to the internal connections and networking of 3D printers. Our design is able to provide PQC encryption while utilizing at most 12% additional overhead in hardware and still maintaining fast message transmission speeds and low message costs for authentication and normal operation. Our design requires 90% and 25% less messages for authentication than existing post-quantum and lightweight CAN solutions, respectively.

Our framework provides significant versatility which can be used to vary the performance and security of the system and structure. First, our design features a customizable subnet size parameter, *k*, which significantly affects framework behavior. We discussed its effect on system hardware efficiency and the impact on “transmission hops”. Second, authentication is performed using both two-way and one-way approaches, which we generally define as router and endpoint authentication, respectively. However, the user may also choose to utilize two-way authentication on endpoints to prove trust in those devices. Finally, we utilize both lightweight and post-quantum cryptographic suites for our design. We did not perform hardware acceleration for either scheme in our results. As a result, an avenue for future work could be testing various quantum-safe and lightweight (or even hybrid) cryptography in software/hardware accelerated packages to produce a comprehensive study in resource-constrained 3D printer applications.

## Figures and Tables

**Table 1:** Comparison of our framework to existing CAN frameworks. As the target application is different, only typical CAN security criteria and security requirements are considered to ensure fair comparison.

	CAN/CAN-FD Frameworks				
Criteria	LWC Framework	PQC Framework	Labrado [12]	Siddiqui [10]	Ravi [11]
Auth Messages	3n	26n	4n	$3n^{2}+2n$	321n
Encryption Type	Lightweight	Post-Quantum	Lightweight	Standard	Post-Quantum
CAN Bus Structure	Tree-based	Tree-based	Direct	Direct	Centralized Router
Secure Private Key	✔	✔	✔	✔	✘
Forward Secrecy	✔	✔	✔	✘	✔
Replay Defense	✔	✔	✔	✘	✔
Plug-and-Play	✔	✔	✘	✘	✘
Message Authenticity	✔	✔	✘	✘	✔
Eavesdropping Defense	✔	✔	✔	✔	✔
Fuzzing Defense	✔	✔	✘	✘	✔
Impersonation Defense	✔	✔	✔	✔	✔
Server Verification	✔	✔	✔	✘	✔
DoS Defense	✦ *	✦ *	✘	✘	✘

✔ - Framework covers criteria, ✘ - Framework does not cover criteria, ✦ - Framework partially covers criteria * Denial-of-service attacks limited to affecting only adversary’s subnet.

**Table 2:** Table of existing 3D printer security solutions and some general features they provide compared to other systems. It should be noted that these systems cannot protect all targets from all cyber-threats, encouraging some combination of these systems to provide strong security protection.

	Three-Dimensional Printing Security Solutions				
Criteria	This Proposal	Beckwith et al. [13]	Safford et al. [20]	Belikovsky et al. [17]	Shi et al. [14]
Security Target	Communication	Print Abnormalities	Printers/Files	Print Abnormalities	File Security
Main Methodology	Tree-based RoT	Machine-Learning	TPM Encryption	Audio Signature	Blockchain
Attack Detection	✔	✔	✔	✔	✘
Post-Attack Detection	✘	✔	✘	✔	✘
Hardware Trojan Defense	✔	✘	✘	✘	✘
Eavesdropping Defense	✔	✘	✔	✘	✔
Tampering Defense	✔	✘	✔	✘	✔
Impersonation Defense	✔	✔	✔	✔	✔
Sensor-based Defense	✘	✔	✘	✔	✘
Print Watermarking	✘	✘	✘	✘	✘

✔ - Framework covers criteria, ✘ - Framework does not cover criteria.

## Data Availability

Data are contained within the article.
